# Alzheimer’s Disease Etiology Hypotheses and Therapeutic Strategies: A Perspective

**DOI:** 10.3390/ijms26146980

**Published:** 2025-07-20

**Authors:** Naomi Scarano, Francesca Musumeci, Beatrice Casini, Chiara Brullo, Pasqualina D’Ursi, Paola Fossa, Silvia Schenone, Elena Cichero

**Affiliations:** 1 Department of Pharmacy, Section of Medicinal Chemistry, School of Medical and Pharmaceutical Sciences, University of Genoa, Viale Benedetto XV, 3, 16132 Genoa, Italy; naomi.scarano@edu.unige.it (N.S.); francesca.musumeci@unige.it (F.M.); beatrice.casini@edu.unige.it (B.C.); chiara.brullo@unige.it (C.B.); paola.fossa@unige.it (P.F.); silvia.schenone@unige.it (S.S.); 2 Institute for Biomedical Technologies, National Research Council, 20054 Segrate, Milan, Italy; pasqualina.dursi@itb.cnr.it

**Keywords:** Alzheimer’s disease, therapeutics strategies, risk factors, FDA approved drugs, non-pharmacological treatments, prevention

## Abstract

Alzheimer’s disease (AD) is a progressive, complex, multifactorial, neurodegenerative disease and accounts for most cases of dementia. The currently approved therapy includes cholinesterase inhibitors, NMDA-receptor antagonists and monoclonal antibodies. However, these medications were gradually discovered to be ineffective in removing the root of AD pathogenesis, having only symptomatic effects. Thus, the priority remains prevention and clarifying AD etiology. A better understanding of the neuroprotective mechanisms undertaken by specific genes is crucial to guide the design of novel therapeutic agents via selective ligands and precision medicine. In this review, we present a perspective of the physiological phase of the AD spectrum, of risk factors in AD with a focus on therapeutic approaches in three categories: neurotransmitters/ion modulations, peptide deposit control and aspecific treatments, followed by a discussion of treatment limitations. An overview of innovative strategies and non-pharmaceutical ancillary support is given.

## 1. Introduction

Alzheimer’s disease (AD) is a neurodegenerative disorder, leading to progressive and irreversible functional and cognitive impairment [1,2,3]. AD represents the prevalent cause of dementia and cognitive impairment in aged people (>65 y/o) [4], and is responsible for 60–80% of dementia cases [5]. In 2021, dementia cases overcame 56 billion people [6], with more than 119,000 deaths officially attributed to AD within only the United States of America [5,7]. AD is prevalently found among the elderly population: only 5% of cases occur before the age of 65 (early onset AD) [8].

AD prevalence further increases after the age of 65: as many as 5% of people in the age range between 65 and 74 have AD. The percentage increases to 13.2% when considering people aged 75 to 84, and 33.4% of people aged 85 or older [5].

**Figure 1:**
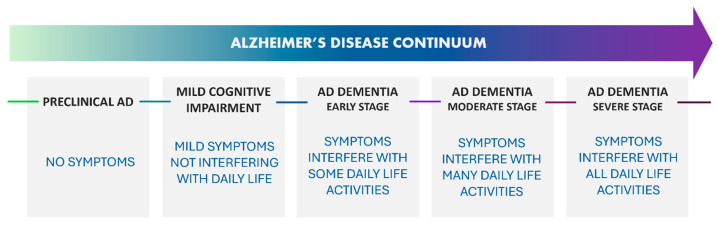
Scheme of the Alzheimer’s disease progression [1].

Several classifications of the advancement stages of the disease were proposed, leading to the definition of several AD stages. However, AD progression is not a discrete process and is referred to as a “continuum” [9]. The classification adopted by the 2024 Alzheimer’s disease facts and figures divides the AD continuum into three phases: (1) preclinical AD, (2) Mild Cognitive Impairment (MCI) phase, and (3) Alzheimer’s dementia. The AD dementia phase can also be sub-divided into mild, moderate, and severe dementia (Figure 1) [5].

During the preclinical phase, individuals do not manifest symptoms such as memory loss or thinking difficulties [10]. However, Alzheimer’s-related brain changes can be observed at this stage, comprehending increased levels of beta-amyloid (Aβ) and Tau protein, decreased metabolism of glucose, and changes in Tau protein in cerebrospinal fluid (CSF) [11,12]. Noticeably, not all patients carrying such modifications develop MCI or dementia before their death, and the ones that result in the development of clinical AD can live for a long time without any symptoms [13].

A combination between fluid biomarkers, imaging, and (more recently) machine learning algorithm can be used to diagnose AD before the development of symptoms [14,15].

The MCI phase instead is characterized by the onset of memory loss, language, and thinking problems. Generally, during this phase, the disease does not interfere with an individual’s daily life. Among MCI patients, about one-third develop Alzheimer’s dementia within five years [16]: 26% of MCI patients, instead, were shown to revert to normal conditions [17].

During the Alzheimer’s dementia phase, the individual experiences degenerative impairment in memory and language properties, and behavioral problems progressively increase their impact on the patient’s daily life [3,5,12]. The early phase generally includes a certain amount of autonomy, but patients may require assistance to perform complex tasks, such as planning, organizing, or making financial decisions. At the moderate AD stage, memory and language impairment are worsened and often accompanied by personality changes, agitation, and suspiciousness. In the last stage of the disease, language skills are severely affected. As a result of damage to brain areas involved in movement, patients are often forced to use a wheelchair or bed [3,12]. Such a condition makes the patients more vulnerable to complications [5,12]: respiratory and urinary infections are among the most common AD-induced co-morbidities.

Depression is frequently observed in AD patients, possibly influencing sleep quality and favoring social withdrawal. In some cases, agitation and delirium can occur. Other AD-derived symptoms or pathologies can include confusion about time and places, problems concerning spatial relationships, changes in personality or mood, urinary incontinence, osteoarthritis, osteoporosis, hearing impairment, epilepsy, and psychosis [12,18,19].

In addition to the complications previously reported, AD often coexists with medical conditions deriving from age or previously existing pathologies [19] such as hypercholesterolemia, hypertension, diabetes mellitus type 2, and atherosclerosis [19,20]. Such co-morbidities can play an important role in AD progression and complicate the scenario for the development of therapeutic opportunities.

## 2. AD Risk Factors, Genetics and Etiology

### 2.1. Risk Factors

**Figure 2:**
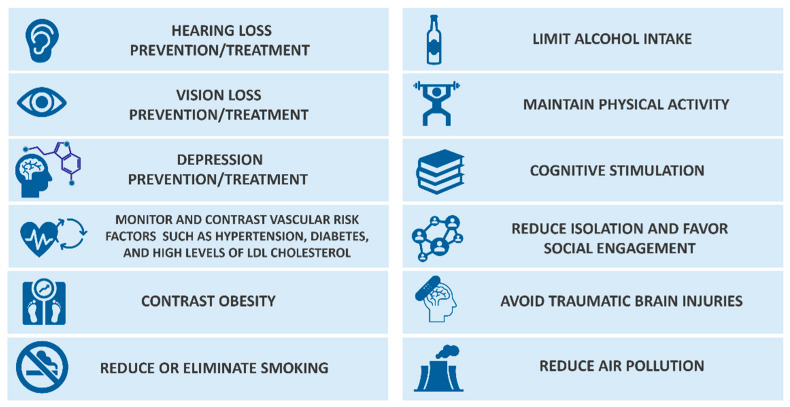
Infographics on the modifiable risk factors for AD development [1] as reported by Lancet [23].

AD is a multifactorial pathology associated with high genotypical and phenotypical heterogeneity [21]. A large amount of risk factors was proposed to be correlated with AD onset, such as pre-existing diseases, lifestyle aspects, psychosocial conditions, and environmental factors [22]. Very recently, a Lancet study proposed an updated list of fourteen modifiable risk factors (Figure 2) which, if correctly treated, would prevent the onset of AD in 50% of cases [23].

The method of study privileged meta-analyses and research comparisons, conferring strong statistical evidence for the presented factors. The list suggests preventing and/or treating (i) hearing loss, (ii) vision loss, (iii) depression, and vascular risk factors such as (iv) hypertension (e.g., via blood pressure control), (v) diabetes, (vi) high levels of LDL cholesterol, and (vii) obesity. Additional suggestions regard lifestyle by showing benefits in (viii) reducing or eliminating smoking, (ix) limiting alcohol intake, and (x) investing in physical activity. Cognitive stimulation (xi) is regarded as a protective factor as well as a reduction in social isolation (xii). Finally, traumatic brain injuries (xiii) should be avoided, and air pollution minimized (xiv).

Along with this, in the literature several non-pharmacological treatments were proposed to alleviate and slow down AD-associated symptoms. Cognitive-oriented training is among the most utilized to reduce functional impairments in patients’ daily life [24].

Physical exercise is an established protective factor for AD development, and when associated with other non-pharmacological treatments such as diet control and cognitive training, an improvement of cognitive parameters was observed [25].

Pro-cognitive effects were highlighted upon dietary modification [26] in AD patients, possibly representing a gut microbiota-mediated effect. Moreover, diets rich in healthy ingredients (fruits, vegetables, whole grains, legumes, nuts, fish, and olive oil) were shown to reduce inflammation, oxidative stress, and insulin resistance. On the contrary, diets with high levels of fat and sugar contribute to higher levels of inflammation and AD biomarkers. Obesity and malnutrition further have a negative influence on AD onset and progression [27]. A multicomponent protocol involving physical, cognitive, social, and other recreational activity exhibits effective reduction in cognitive impairment in MCI and moderate stages of dementia [28]**.** Noticeably, several clinical cases reported reverting cognitive impairment or cognitive amelioration especially in the first phases of AD when treatment comprehended functional and lifestyle intervention [29,30,31].

The suggested modifications to prevent AD should be implemented as early as possible and maintained over time. On the contrary, advanced age, family history, as well as several genetic factors are regarded as non-modifiable risk factors for AD development [32,33].

### 2.2. Genetics

Around 0.1% of AD cases are caused by genetic inheritance, leading to early development of AD symptoms (30–50 y/o) [34]. The most studied mutations are related to Presenilin 1 (*PSEN1*), Presenilin 2 (*PSEN2*), and Amyloid precursor protein (*APP*) genes, all influencing the Aβ_42_ formation [35]. Mutation of PSEN1 leads to severe AD forms appearing even at 25 y/o [32]. Mutations in the PSEN2 exhibit incomplete penetrance, sometimes failing to lead to its pathology, and the onset is later with respect to the PSEN1 cases [32].

Regarding the APP gene, 32 pathogenic mutations were identified [32], reported to increase the Aβ42/40 ratio, total Tau quantity, and its phosphorylation [32,36,37]. Additionally, Down Syndrome is regarded as a further risk factor for AD development due to the location of the APP gene on chromosome 21 [38]. Its triplication in Down Syndrome patients leads to APP overexpression and an increased production of Aβ peptides [38]. Polymorphisms of the apo-lipoprotein such as ε3 or ApoE-ε4 also may influence the risk of developing AD [39]. Mechanistically, ApoE-ε4 binds to Aβ weakening Aβ clearance [40]. Additionally, ApoE-ε4 is less efficient than other ApoE isoforms at promoting the degradation of soluble Aβ within microglia [41]. Several other genes were associated with an enhanced risk of developing AD or were proposed to have a role in AD pathogenesis [32] (e.g., the Triggering receptor expressed on myeloid cells 2 (TREM2) [42], the ATP-binding cassette subfamily A member 7 (ABCA7) [43], the Bridging integrator 1 (BIN1) [44], etc.).

### 2.3. Etiology

AD is considered a multifactorial disease, in which complex interactions among different processes occur. Beyond genetic factors, other hypotheses were formulated, such as the presence of the Tau Disease Protein 43 (TDP-43) pathology [45], deriving from the accumulation of truncated/phosphorylated forms of the nuclear protein TPD-43, Vitamin B5 deficiency [46], Fyn kinase overexpression [47], cyclin dependent kinase 5 (Cdk5) hyperactivation [48], and many others.

**Figure 3:**
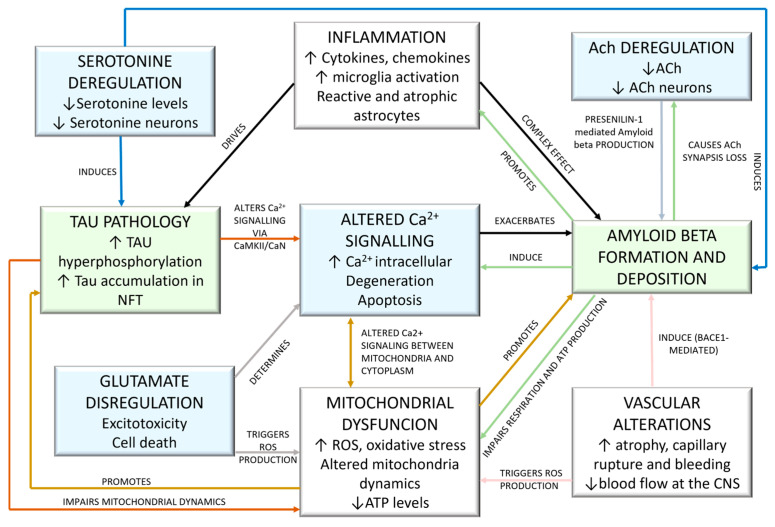
The most relevant hypotheses on AD etiology [1]. Neurotransmitters/ion-based, peptide-based and aspecific hypotheses are highlighted in cyan, green, and gray.

The most relevant AD etiology hypotheses [34,49] and have been deeply explored in drug design include mechanisms influencing AD pathogenesis, such as neurotransmitter- or ion-based hypotheses, peptide-based hypotheses, and aspecific pathways (Figure 3).

Among the neurotransmitter-based hypotheses, the serotonine-based one is interconnected with the amyloid beta and Tau pathologies, while ACh level depletion turns into Presenilin-1 (PS-1) mediated amyloid beta production. In addition, compromised ACh levels directly correlate with cognitive impairment. Glutamate dysregulation is strictly related to cellular toxicity and stress events, causing altered calcium signaling and mitochondrial dysfunction. Altered calcium signaling induces mitochondrial dysfunction and oxidative stress, acting also at the amyloid beta formation. Among the peptide-based hypotheses, the amyloid-based one is strictly interconnected with most of what has been previously mentioned, impairing neurotransmitters systems such as the ACh one (directly) and the glutamate one via altered calcium signaling. Tau pathologies are mainly involved in mitochondrial dysfunction. Aspecific events, such as neuroinflammation and vascular alterations, participate in both the two peptide-based hypotheses. As a result, as a preventive approach, inflammation control and putative vascular disease interventions could contribute to reducing amyloid beta formation. Care in serotoninergic-based prescriptions could be of help in preventing AD-associated pathologies. More details are described in the following sections.

## 3. Neurotransmitter- and Ion-Based AD Hypotheses and Therapeutics

### 3.1. Cholinergic Hypothesis and Therapeutics

#### 3.1.1. Cholinergic Hypothesis

Acetylcholine (Ach) binds to the cholinergic receptors helping learning, synaptic plasticity, and memory functions [50]. The central role of ACh in the cholinergic hypothesis was due to the observation of a significant loss of cholinergic neurons in the brains of AD patients, accompanied by a lowering of ACh levels [51]. This turns into AD-related mechanisms, such as neuroinflammation [52]. Additionally, reduction in the activity of the enzyme responsible for ACh synthesis (choline acetyltransferase, ChAT) correlates with cognitive impairment observed in AD [53]. Inhibiting ACh esterase (AChE), a hydrolase which catalyzes the hydrolysis of acetylcholine to choline and acetate, contributes to preventing ACh decrease at the synaptic cleft. In addition, AChE enhances presenilin-1 (PS-1) expression [54], a subunit of the gamma-secretase complex with catalytic activity positively correlated with the production of Amyloid β. Conversely, Amyloid β seems to cause cholinergic synaptic loss and impairs ACh release [12]. As a consequence, inhibiting AChE can also be evaluated to limit PS-1 mediated Amyloid β production.

AChE was targeted by several drug discovery campaigns, resulting in the approval of four drugs for the treatment of AD (**donepezil**, **rivastigmine**, **galantamine**, **tacrine**), as AChE inhibitors (AChEIs) [55].

#### 3.1.2. Cholinergic-Based Therapeutics

Significant research efforts were employed in targeting the cholinergic hypothesis. To improve cholinergic transmission, the concomitant use of AChEIs and Ach precursors was proposed. An example is given by the use of **donepezil** and **choline alphoscerate**, evaluated in clinical trials. Behavioral and cognitive improvements were highlighted in AD patients [56,57].

**Figure 4:**
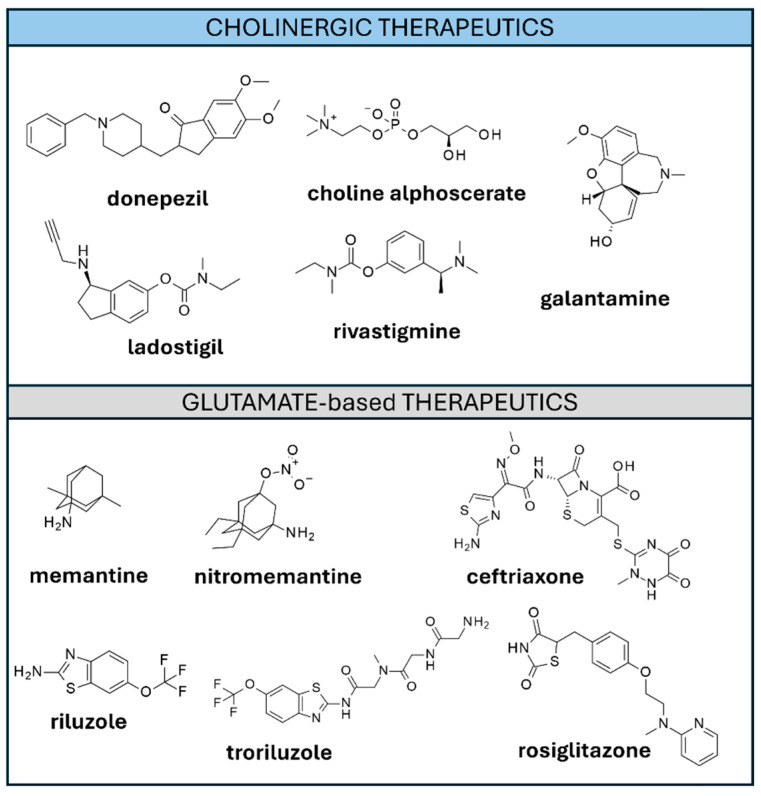
Chemical structure of cholinergic- and glutamate-based therapeutics exploited in AD [1].

The most explored design of AChEIs led to the approval of the aforementioned **donepezil,** and of **rivastigmine** and **galantamine** (see Figure 4).

Additionally, there have been attempts to combine the pro-cholinergic dual inhibition action of AChE and other enzymatic targets with other favorable pharmacological effects, such as the hybrid AChE/MAO-B inhibitor **Ladostigil** (Figure 4) [58]. Clinical tests of **Ladostigil** showed its good tolerability but lack of efficacy in slowing down AD progression [59] (NCT01429623). The same strategy was applied to the design of dual AChE/GSK-3β inhibitors [60,61] and dual AChE/PDE4D [62].

Furthermore, the design of allosteric modulators on AChR subtypes [63,64,65,66] was explored. α7 nAChR positive allosteric modulators exhibited a promising pro-cognitive effect in animal models [67,68,69,70]. Clinical studies highlighted the unclear efficacy of this class of drug candidates [71]. M1-mAChRs were also explored in this perspective, in some cases reaching early clinical experimentation [72]. Nerve Growth Factor administration was attempted to contrast the degeneration and loss of cholinergic neurons; moderate success was achieved [73].

### 3.2. Glutamate Excitotoxicity Hypothesis and Therapeutics

#### 3.2.1. Glutamate-Based Hypothesis

Glutamate (L-glutamic acid) is an important excitatory neurotransmitter involved in learning and memory and hypothesized to be at the basis of AD-inducing processes [74]. Dysregulation in the glutamatergic system leading to excessive extracellular concentrations of glutamate was shown to cause neurotoxicity (excitotoxicity), inducing nervous cell death [75]. According to a recent meta-analysis, the glutamatergic system is significantly altered in AD patients [76]. The altered expression of the glutamatergic receptor and transport protein was also observed in sporadic AD [77]. The excitotoxic effect seems to be exerted through an overstimulation of the post synaptic response, which causes a major entrance of calcium into neurons [78]. The consequences of abnormal calcium increase in cytoplasm are Reactive Oxygen Species (ROS) production increase, cellular collapse via cytoskeletal damage and membrane disruption, and tau hyperphosphorylation induction via kinase stimulation [79]. Proposed therapies related to the glutamate hypothesis comprehend different mechanisms of action, such as the modulation of glutamate transporter-1 (GLT-1) or the blocking of the N-methyl-D-aspartate receptor (NMDAR).

#### 3.2.2. Glutamate-Based Therapeutics

The glutamate excitotoxicity hypothesis received significant attention over the years, and its study resulted in the approval of the selective E-NMDAR antagonist **memantine** (Figure 4). **Memantine** exerts a moderate pro-cognitive effect in moderate-to-severe AD. Lower efficacy was observed in earlier AD stages [80]. A modified version of **memantine**, namely **nitromemantine** [81], combined the affinity of E-NMDARs with the introduction of a nitro group acting as a NO/redox modulator. Preclinical studies underline a possible improved effect with respect to **memantine** [82].

Another strategy to develop AD treatments involves the upregulation of glutamate transporters such as Glutamate Transporter-1 (GLT-1). Such macromolecules, in fact, remove glutamate from the synaptic cleft after glutamate release, reducing its excess and subsequently its excitotoxicity [83]. In aging and AD, the number of glutamate transporters is significantly diminished [84].

A few examples of drugs activating/upregulating GLT-1 are **Ceftriaxone**, **Riluzole** (Figure 4), corticosteroids, **estrogen**, and **insuline**, among others. **Ceftriaxone** is an antibiotic upregulating GLT-1 expression. In preclinical models, a pro-cognitive effect and an amelioration of the AD neuropathological scenario were observed [85]. In other ones, **ceftriaxone** was shown to impair synaptic plasticity and memory recognition [86]. **Riluzole**, a drug in use for the treatment of Lateral Amyotrophic Sclerosis, was repurposed for AD as it increases glutamate uptake via several glutamate transporters (GLT-1, GLAST, EAAC1) [87].

Phase II clinical test involving **riluzole** (NCT01703117) highlighted poor pharmacokinetics, leading to the design of a **riluzole** analog (**troriluzole**) recently evaluated in a phase II/III clinical trial (NCT03605667). Unfortunately, lack of efficacy was reported upon trial completion [88]. Corticosteroids upregulate GLT-1 via post-translational modification [89] and were shown to reduce Aβ levels in cerebrospinal fluid of AD patients (NCT00912886). **Estrogen** increases GLT-1 and glutamate aspartate transporter (GLAST) expression, enhancing glutamate uptake [90]. Clinical studies, however, did not confirm the expected benefits [91]. Accordingly, administration of the antidiabetic **Rosiglitazone**, associated with glutamate uptake regulation via GLT-1 upregulation [92], resulted in poor efficacy outcomes [93,94]. Finally, animal models highlighted that insulin-induced increase of sodium-dependent glutamate uptake occurs in a way independent of GLT-1 [95]. Nasal **insulin** administration ameliorated memory and function in daily life in several clinical studies [96] (NCT01547169, NCT01595646, NCT00438568). Other clinical studies, however, reported less encouraging results [97] (NCT01767909).

### 3.3. Serotoninergic Hypothesis and Therapeutics

#### 3.3.1. Serotoninergic Hypothesis

The serotoninergic hypothesis was born from the observation of large neuron loss in the serotonergic nucleus raphe dorsalis (NRD) in AD patients [98]. Moreover, low levels of serotonin (5-HT) [99,100] were observed in early AD tissue analysis, and correlations were observed between cognitive decline and the loss of serotonin receptors (5-HTRs) such as 5-HT_2A_ [101]. Preclinical studies highlighted the linkage between some 5-HTRs and AD hallmarks such as Aβ deposition and tau hyperphosphorylation in animal models and cells [102,103,104,105].

Different 5-HTRs are known and often specifically expressed in brain areas devoted to different functions. However, each of the 5-HTRs contributes in different manners to promote/prevent AD progression, and their pharmacological modulation needs careful evaluation. An example is the 5-HT_4_ receptor, whose agonism represents a protective factor for AD [106,107]; in contrast, the 5-HT_6_ receptor was proven to have a precognitive role when antagonized [108,109]. Nevertheless, the mechanisms of the 5-HTRs influence on cognition are not completely clear. On the other hand, well known serotonin-related diseases frequently appear as co-morbidities of AD [110]. Behavioral and Psychological Symptoms of Dementia (BPSD), comprehending cognitive and memory impairment [111], depression [112], hyperactivity [113], apathy [114], and psychosis [115], were connected to serotoninergic dysfunctions [110]. Based on the above, optimization of serotoninergic targeting strategies would require further investigation to elucidate in detail the connection between the serotoninergic system and AD pathogenesis.

#### 3.3.2. Serotoninergic-Based Therapeutics

**Figure 5:**
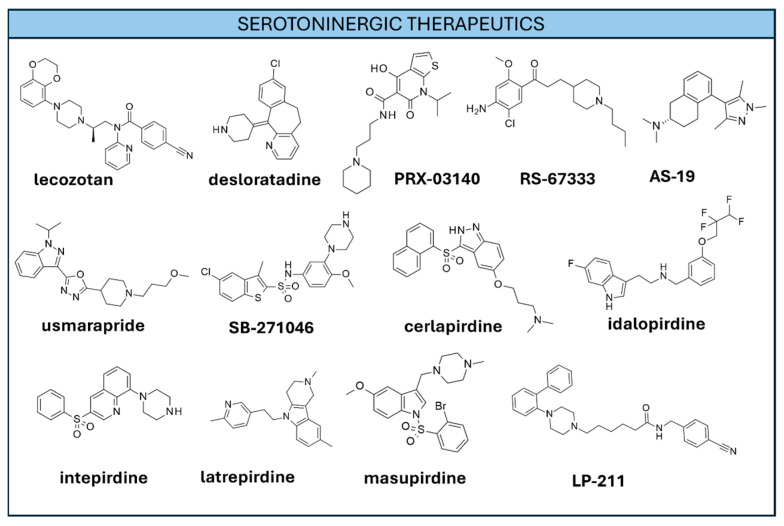
Chemical structure of serotoninergic therapeutics exploited in AD [1].

Several drugs targeting serotoninergic receptors are currently used to treat neuropsychiatric symptoms associated with AD. 5-HT_1A_R antagonists such as **Lecozotan** (Figure 5) were shown to enhance cognition in animal models [116]. Unfortunately, clinical trials did not confirm the efficacy of such compounds [117] (NCT00151398, NCT00277810).

The antiallergic drug **desloratadine** (Figure 5) was tested against the 5-HT2_A_ receptor in a repositioning perspective and was shown to ameliorate AD pathology in animal models [118]. Other animal studies highlighted the potential of 5-HT2_A_ antagonists in the AD field [119].

The chronic administration of the 5-HT_4_R partial agonist **RS-67333** (Figure 5) to 5XFAD mice was shown to ameliorate amyloid pathology and neuroinflammation as well as cognitive aspects [120]. Early administration, instead, prevents the onset of cognitive impairment and behavioral symptoms [121]. The compound was also tested in animal models in combination with **galantamine**, exhibiting the ability to reverse cognitive impairment and better management of adverse effects [122]. Another 5-HT_4_R partial agonist, **PRX-03140** (Figure 5), was shown to exert a promnesic effect and enhance visuospatial memory in animal models, possibly by elevating ACh and brain-derived neurotrophic factors [123]. Despite the encouraging results deriving from early clinical trials, **PRX-03140** advanced experimentation was discontinued (NCT00384423, NCT00672945) [124]. More recently, other 5-HT_4_R partial agonists such as **Usmarapride** (**SUVN-D4010**) (Figure 5) [106] were developed. **Usmarapride** phase I clinical evaluation highlighted a satisfying safety, tolerability, and PK profile [125].

5-HT_6_R antagonists were submitted to extensive investigation in the field of AD. Several compounds belonging to this class were observed to exert promising pro-cognitive effects in preclinical and early clinical trials [126,127,128]. **SB-271046** (Figure 5) was shown to exert a favorable action on dopaminergic neurons in animal models [129]. **Cerlapirdine** (Figure 5) is a potent 5-HT_6_ receptor antagonist also targeting 5-HT_7_ and 5-HT_2B_ receptors [130]. Early clinical studies exhibited a moderate pro-cognitive effect as well as a good tolerability profile [131]. **Idalopirdine** (Figure 5) is a 5-HT_6_ receptor antagonist, with additional affinity for adrenergic receptors (α_1A_ and α_1B_) and (to a minor extent) for 5-HT_2A_ and 5-HT_2C_ receptors [132]. It was shown to potentiate the pro-cognitive effect of **donepezil** in animal models [133]. However, advanced clinical trials failed in demonstrating the pro-cognitive effect of **idalopirdine** [134] and of the other 5-HT_6_ antagonists **Intepirdine** (Figure 5) [135], **Latrepirdine** (Figure 5) (NCT00675623, NCT00829374), and **Masupirdine** (Figure 5) [136]. Regarding the 5-HT_7_R targeting compounds, the selective 5-HT_7_R agonist **AS-19** (Figure 5) was shown to improve cognition and memory in animal models [137,138]. The 5-HT_7_R agonist, **LP-211** (Figure 5), was shown to preserve neurons from Aβ-induced damage as well as avoid cognitive impairment in preclinical studies [139].

### 3.4. Calcium Signaling Hypothesis and Therapeutics

#### 3.4.1. Calcium Signaling Hypothesis

Ca^2+^ ion is present in the neuronal cytoplasm and other organelles, and it has several roles in synaptic plasticity, neurotransmitter transmission, learning ability, and memory. The calcium signaling hypothesis individuates the dysregulation of calcium homeostasis induced by the activation of the amyloidogenic pathway as a critical factor in AD development [140,141]. Several studies documented that disruption of Ca^2+^ regulation causes degeneration, apoptosis, autophagy deficit, and abnormal neuronal plasticity [142]. Ca^2+^ signaling disturbance resulting in increased concentration of Ca^2+^ in the cytoplasm may exacerbate Aβ deposition and tau hyperphosphorylation, leading to the formation of a vicious circle [49].

Conversely, Ca^2+^ signaling is altered by the Tau protein hyperphosphorylation [142]. The interplay between calcium signaling and Tau phosphorylation is linked to Ca^2+^/Calmodulin-dependent protein kinase II (CaMKII) and calcineurin (CaN), two proteins involved in calcium signaling. CaMKII is involved in Tau phosphorylation, while calcineurin is involved in Tau dephosphorylation. Altered expression levels or dysregulation of the CaMKII/CaN system lead to calcium imbalance and abnormal Tau phosphorylation [143,144]. Aβ oligomers, instead, can increase calcium concentration through different mechanisms, including the formation of membrane pores [145], NMDA receptors activation [146], voltage-gated calcium channels (VGCCs) [147] regulation, and metabotropic glutamate receptor 5 (mGluR5) activation [148]. Additionally, cytoplasmic calcium level increase can result from the Aβ-induced release of Ca^2+^ from the Endoplasmic Reticulum (ER) through calcium channel receptors, such as ryanodine receptors (RyRs) and inositol 1,4,5-trisphosphate receptors (IP3Rs) [142]. In addition to ER, mitochondrial Ca^2+^ homeostasis was proposed to be involved in AD pathogenesis [49,149]. In neurons, mitochondria are particularly needed at the synapse, where they produce a buffer Ca^2+^ concentration as well as ATP, inducing the formation of the membrane potential along the axon to allow neurotransmission. In AD patients, mitochondrial function, as well as ATP levels and Ca^2+^ buffering [150,151], is shown to be reduced.

#### 3.4.2. Calcium-Signaling Modulators

**Figure 6:**
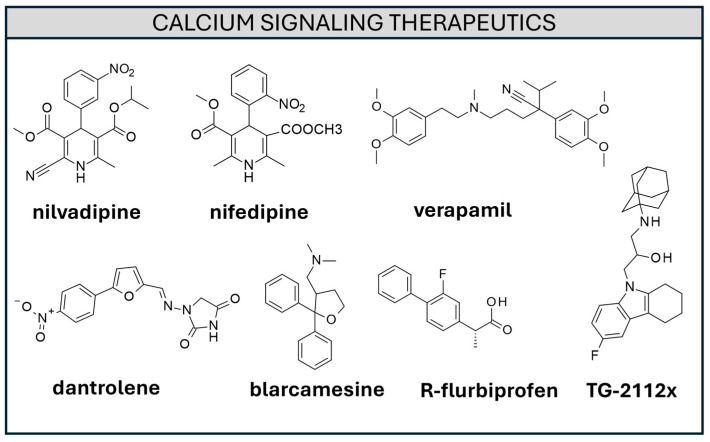
Chemical structure of calcium-signaling modulators exploited in AD [1].

Different strategies were applied to target the calcium signaling hypothesis, involving the regulation of cytosolic, ER, and mitochondrial Ca^2+^ levels [152]. Both the voltage-gated Ca^2+^channels (VGCCs) [153] (allowing Ca^2+^ entrance upon neuronal depolarization) and receptor-operated Ca^2+^ channels (ROCs) (subsequently enabling calcium influx to the binding of a proper agonist) were targeted. A few examples of VGCC inhibitors are represented by **nilvadipine**, **nifedipine**, and **verapamil** (Figure 6) [152].

The most advanced of the class is **Nilvadipine**. Its phase III clinical evaluation highlighted a good safety profile, but a lack of efficacy in slowing down cognitive impairment [154]. Among the ROCs class, the NMDARs represent a subfamily of relevance in the field of AD: NMDAR blockage and were shown to reduce abnormal AD-related calcium influx [155]. The FDA-approved drug **Memantine** belongs to the NMDARs blocker class, restricting Ca^2+^ influx, reducing excitotoxicity as well as maintaining physiological functionality of the receptor. (see par. 5). AChEIs as well as the monoclonal antibody **aducanumab** (see Paragraph 4.3) are hypothesized to exert their action also by involving Ca^2+^ signaling [156,157,158,159]. ER calcium pumps such as 1,4,5-trisphosphate receptors (IP_3_Rs) [160] or ryanodine receptors (RyRs) [161] are considered targets of relevance for AD. In 2017, an in vivo study explored the potential of the RyR receptor as a drug target for AD, hypothesizing a leak of such receptors in the presence of the pathology. A RyR/clastabin2 stabilizer (Rycal) was able to reverse this leak, inducing pro-cognitive effects and behavioral benefits in animal models [162]. Moreover, a negative allosteric modulator (NAM) of RyR (**Ryanodex as dantrolene sodium**) was shown to reduce Aβ load, rescue synaptic plasticity, and normalize ER Ca^2+^ signaling in animal models [163,164,165].

Nonsteroidal anti-inflammatory drugs such as **Salicylate** and **(R)-Flurbiprofene** (Figure 6) were shown to mildly depolarize mitochondria, inhibiting Ca^2+^ mitochondrial uptake without altering cytosolic Ca^2+^ levels [166]. The novel compound **TG-2112x** (Figure 6) [167] was shown to inhibit mitochondrial Ca^2+^ overload and was shown to exert a neuroprotective action against glutamate excitotoxicity. The compound that was evaluated exhibited neuroprotective potential from β-amyloid-induced cell death in animal models [168]. **Anavex 2-73** (**blarcamesine**) (Figure 6) [169] is a small molecule activating the sigma 1 receptor (S1R), a receptor expressed on mitochondria-associated endoplasmic reticulum membranes. S1Rs activation was found to be involved in synaptic plasticity and neuroprotection and was downregulated in AD [170]. **Blarcamesine** has recently completed a clinical phase 2a/3 trial with encouraging results [171,172]. On this basis, further efforts in the search for selective S1R agonists could be attempted, in order to enhance synaptic plasticity and to design neuroprotective agents.

## 4. Peptide-Based AD Hypotheses and Therapeutics

### 4.1. Amyloid Deposits Hypotheses and Therapeutics

#### 4.1.1. Amyloid Hypothesis (Amyloid Cascade)

The amyloid hypothesis individuates the deposition of Aβ peptide in the brain parenchyma as the cause of AD development [173]. Additionally, the hypothesis requires that in AD conditions, the normal clearance of Aβ is disrupted. Aβ is derived from the Amyloid Precursor Protein (APP), a transmembrane protein, which is cleaved by alpha- and gamma-secretases. The APP gene mutation was individuated as the cause of early onset and familiar forms of the pathology [174], as well as other Aβ-related genes (PSEN1/2).

Such cleavage produces several peptides of different lengths. Of particular interest is the formation of Aβ_40_ and Aβ_42_ (40 and 42 amino acid peptides, respectively). Aβ_40_ is a soluble form of Aβ, while Aβ_42_ (42 amino acids peptide) is less soluble and tends to aggregate [12,34]. An abnormal production of Aβ_42_ leads to the formation of senile plaques and was supposed to trigger a cascade of processes such as inflammation, oxidative stress, and Tau protein accumulation, leading to neuronal loss and finally to AD symptoms [49]. The amyloid cascade hypothesis is supported by the direct observation of Aβ plaques in the brain tissues of AD patients [175,176].

Genetic variation of the apo-lipoprotein-E (ApoE), a protein involved in the clearance of Aβ, is identified as a strong risk factor leading to the AD development [177]. Transgenic mice were extensively used to prove the pro-cognitive efficacy of pharmacological, genetic, and immunological interventions in reducing Aβ load [178,179,180]. However, several criticisms of the amyloid cascade hypothesis were continuously formulated [181]. In particular, the correlation between Aβ plaques and AD symptoms is not well assessed; on the contrary, it is possible to observe Aβ deposition without any sign of cognitive impairment [182]. More recently, the cause of the disease was switched to soluble forms of Aβ, better correlating with AD symptomatology and cognitive impairment with respect to plaques [183].

#### 4.1.2. Aβ-Targeted Therapeutics

**Figure 7:**
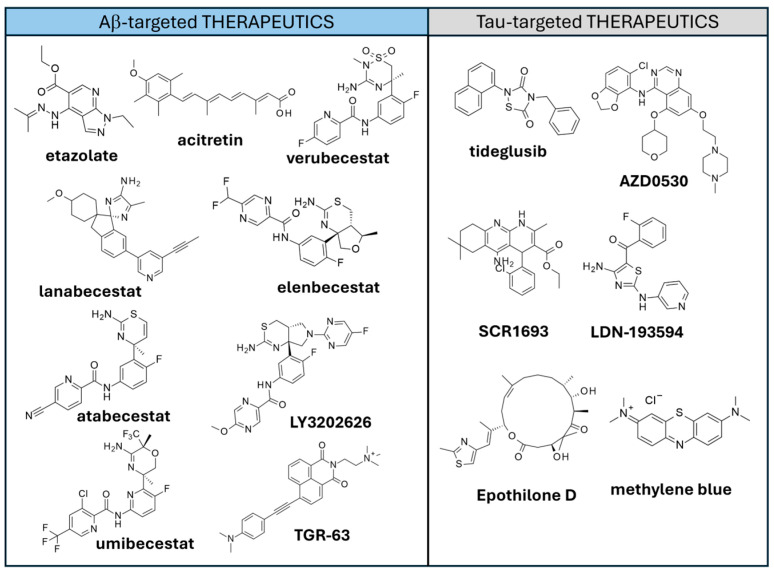
Chemical structure of peptide-based deposit modulators exploited in AD [1].

Amyloid-targeted treatments addressed both the modulation of APP cleavage by means of secretases [184] and Aβ aggregation inhibition [185]. The three classes of secretases (α, β, γ) were targeted. α-secretases are involved in the non-amyloidogenic pathway of Aβ cleavage and activators were designed to favor this pathway. A few examples of α-secretase activators reaching clinical evaluation are **Etazolate** and **Acitretin** (Figure 7). **Etazolate (EHT0202**) exhibited safety and tolerability, but the pilot study did not highlight significant improvement in cognitive performance upon drug administration [186]. **Acitretin** was proven to elevate sAPP levels in CSF and lower the Aβ levels in early clinical studies [187]. β-secretases are involved in the cleavage of APP, leading to the formation of Aβ. Inhibition of β-secretases 1 (BACE1) is thus expected to decrease amyloid beta production (and deposition) [188]. BACE1 inhibitors such as **Verubecestat**, **Lanabecestat**, **Elenbecestat**, **atabecestat**, **LY3202626**, and **Umibecestat** (Figure 7) exhibited promising effects on preclinical and phase I–II clinical trials, but further clinical studies revealed a lack of efficacy and/or significant adverse effects [188].

Then, γ-secretases are also involved in APP cleavage, representing a promising target for the anti-amyloid strategy [189]. However, γ-secretases inhibitors were shown to exert important off-target effects (e.g., due to Notch signaling alteration) [190] and toxicity issues [191], causing the failure of clinical trials. The development of allosteric γ-secretases inhibitors may lead to the revaluation of this therapeutic strategy [192].

Peptides and peptidomimetics targeting Aβ were also developed to inhibit Aβ aggregation [193,194]. In some cases, peptidomimetics were proven to stabilize the monomeric form of Aβ as well as reduce ROS generation [195]. Furthermore, small molecules such as **TGR-63** (Figure 7) were evaluated in vitro and were shown to possess anti-aggregation potential towards Aβ aggregates [196]. Aβ load reduction and ameliorated memory, as well as learning and cognitive parameters were highlighted in mouse models [196].

Immunotherapy approaches were also applied, including active immunization (through vaccines developed on the antigen peptide) and passive immunization (monoclonal antibodies directly targeting the undesired protein), not leading to encouraging results as cognitive improvement [197].

### 4.2. Tau Hypothesis and Therapeutics

#### 4.2.1. Tau Hypothesis

In addition to Aβ plaques, the formation of neurofibrillary tangles is regarded as a major hallmark of AD development [176]. Neurofibrillary tangles (NFTs) are intracytoplasmic fibrils formed by the aggregation of hyperphosphorylated Tau protein, a phosphoprotein involved in the stabilization of microtubules. The Tau hypothesis [198] individuates, in the formation of neurofibrillary tangles, the cause of the loss of neuron functionality and subsequent cognitive impairment associated with AD. Their presence is related to cognitive impairment symptoms [199,200,201], and their number and distribution are strongly correlated with the severity of such effects [202]. Genetic evidence highlights the importance of the mutation of the Tau gene in the development of neurodegeneration [203].

The interplay between amyloid and Tau processes in the AD pathogenesis was hypothesized to be of the “trigger and bullet” type, with amyloid initiating the pathogenic process (trigger), and Tau provoking the NFTs formation, inducing neurodegeneration (bullet) [12,204]. However, such a hypothesis was confuted as NFTs are observed before the comparison of Aβ aggregates [205,206,207]. Moreover, Tau pathology was shown to be independent from the Aβ mechanism, leading to the conclusion that Aβ clearing strategies may not impair Tau pathology [208]. However, other studies highlight the mutual influence between Tau and Aβ mechanisms, hypothesizing a synergistic effect between the two processes [209,210]. Tau pathology was further shown to interact with other mechanisms such as microglia activation, which was shown to drive tau pathology onset and progression in animal models [211]. Further limitations include the fact that Tau abnormalities can be found in several other neurodegenerative diseases, such as progressive supranuclear palsy and corticobasal degeneration, highlighting its non-specificity to AD [212].

#### 4.2.2. Tau-Targeted Therapies

A possible strategy to target Tau pathology is to correct abnormal tau phosphorylation. Several relevant kinases were considered in this perspective. Among them, the GSK-3β is among the most studied, leading to the development of several inhibitors over the years. One of them, **Tideglusib** (Figure 7), reached clinical trial with a good safety profile; unfortunately, **Tideglusib** lacked therapeutic efficacy [213]. Fyn kinase inhibitor **AZD0530** was evaluated for AD in clinical trials, unfortunately, with no effects on cognition decline [214]. Isoform selective JNK3 kinase inhibitors were designed and evaluated in mouse models, highlighting ameliorated cognitive performances [215]. A hybrid between an AChE inhibitor and a Calcium channel blocker (**SCR1693**; Figure 7) was shown to promote tau dephosphorylation as well as Aβ reduction in vitro [216]. Multi-kinase inhibitors were also evaluated, such as the dual CDK5 and GSK-3β inhibitor **LDN-193594**. Animal model studies highlighted its efficacy in the treatment of cognitive impairment and reduction in hyperphosphorylated tau levels [217]. Recently, hyperphosphorylated tau degradation was attempted through the use of dephosphorylation targeting chimera (DEPTAC), which are molecules that promote the molecular interaction between tau and phosphatase [218]. As a result, pTau dephosphorylation is enhanced.

In addition to targeting post-translational modifications, a tau-related strategy aimed at enhancing microtubule stabilization was developed [219]. Several cancer therapies were proven to exert microtubule stabilization and were then repurposed for AD treatment [219,220]. As an example, **Epothilone D** (Figure 7) was tested in a mouse model, and exhibited enhancement of microtubule density, resulting in proper axonal transport and improved cognitive outcomes [221,222,223].

Small molecules such as **methylene blue (**Figure 7) [224] and related analogs [225,226] proved to inhibit tau aggregation in vitro.

Regarding immunotherapy, a few vaccines were developed on pathogenic fragments and phosphorylated tau. **AADvac1** [227], targeting a sequence involved in pathogenic tau–tau interaction, was effective in mouse models in reducing tau and Aβ burden, as well as microglia activation [228] even if ineffective in enhancing cognition levels in clinical trials [229]. **AV-1980D**, a DNA-based vaccine targeting tau N-terminus, reduced Tau burden in animal models [230]. Passive immunization was also explored, with the design of several monoclonal anti-tau antibodies (Abs). **ABBV-8E12/Tilavonemab** [231] and **Gosuranemab** [232], two monoclonal Abs (mAbs) with a tau clearing function, exhibited promising Tau clearance and reversal of cognitive impairment in mouse models. Early clinical studies highlighted good tolerability [233,234]; however, clinical evaluation (NCT02880956) was discontinued [234] due to a lack of pro-cognitive efficacy. Currently, the phase III clinical trial of **E2814** (**Etalanetug**) is ongoing [235].

The PROTAC technology was also applied to target tau proteins, with the aim of reducing tau levels. A chimeric peptide composed of a peptidic tau-recognizing sequence and a second sequence binding E3 ligase was combined with a third segment for improved cell permeability. The corresponding candidate was evaluated in AD mouse models, showing efficacy in lowering tau levels [236]. More recently, a small molecule PROTAC was designed with the same purpose, exhibiting efficient tau clearance and significant pro-cognitive effects in animal models [237].

## 5. AD Aspecific Hypotheses and Therapeutics

### 5.1. Neuroinflammation-Based Hypotheses and Therapeutics

#### 5.1.1. Neuroinflammation

AD pathology is associated with higher levels of inflammation markers such as cytokines and chemokines in the CNS [238,239], and pathologies associated with chronic inflammatory states such as obesity [12,240] or diabetes [240] are risk factors for the development of AD. Of particular interest for the analysis of neuroinflammation in AD is the role of microglia, a type of glia cell devoted to the immune response in the CNS [241]. Microglia, when activated, is able to release cytokines, chemokines, free radicals, and different inflammatory responses. In addition, the microglia cells have a clearance function, being involved in the apoptosis induction of damaged neurons and plaques. Accordingly, microglia seem to exert a dual role on AD [241].

It has been suggested that microglia activation in early phases of AD may be beneficial for Aβ clearance, but at later stages it induces inflammation and apoptosis [34,241,242]. Similar considerations can be made for astrocytes, which are glial cells involved in brain homeostasis maintenance, GABA system regulation and metabolism, BBB support, and ionic concentration regulation, among others [243,244]. In AD conditions, astrocytes were found in different states, including both reactive astrocytes and atrophic astrocytes [244]. Reactive astrogliosis possesses a neuroprotective role by favoring Aβ clearance, releasing transforming growth factor β (TGF-β), supporting homeostasis, phagocytosing sub-functional neurons, and intervening to synthesize and release GABA to contrast neuron hyperexcitability. In contrast, reactive astroglia can induce neuronal damage, as well as the release of excessive quantities of complement protein C3 and hydrogen peroxide [244]. Moreover, astrocytic atrophy associated with impaired astrocyte function was shown to lead to impaired homeostatic support and blood–brain barrier (BBB) integrity disruption, as well as synaptic hyperexcitability [244]. Non-neuronal cells, such as endothelial cells, can also contribute to the release of pro-inflammatory agents. In the brain blood vessels of AD patients, for example, the release of IL-6, IL-1b, and TNF-alpha was enhanced with respect to healthy people [245].

#### 5.1.2. Therapeutics for Neuroinflammation

**Figure 8:**
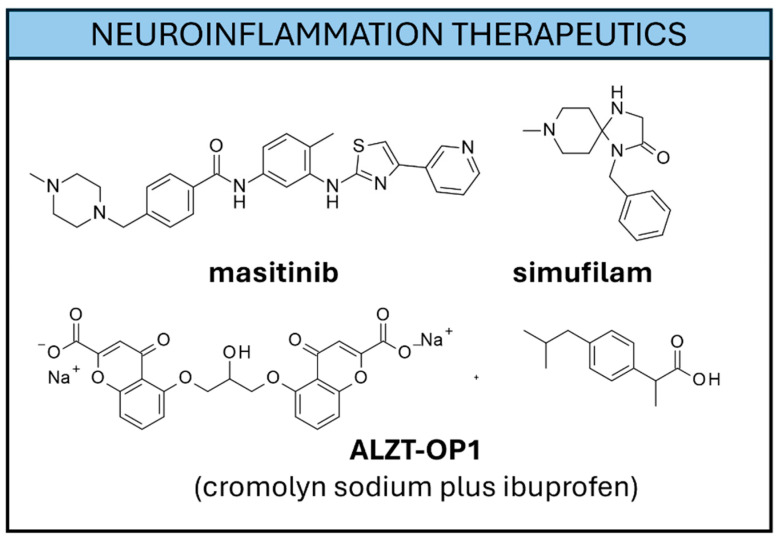
Chemical structure of neuroinflammation modulators exploited in AD [1].

Beyond natural compounds as potential anti-inflammatory agents in AD, synthetic small molecules have been explored. Among them, the selective tyrosine kinase inhibitor **Masitinib** (Figure 8) [246] was developed to contrast AD-associated neuroinflammation.

**Masitinib** mechanism of action is exerted via the mast cells, a type of immune system cell in the brain. Phase II/III clinical trials (NCT01872598) highlighted a certain potential in slowing down AD progression in mild-to-moderate AD patients [247]. A clinical evaluation of **Masitinib** as adjunct to AChEIs and/or **memantine** is scheduled and expected to be completed in 2026 (NCT05564169). **Simufilam** [248] is a small molecule acting on neuroinflammation by targeting an incorrect form of filamin A occurring in AD pathology. This event corrects the aberrant interaction of filamin A with other Ach and chemokine receptors, reducing the release of inflammation chemokines [248] by Aβ-stimulated astrocytes. The phase IIa clinical trial highlights the reduction in biomarkers associated with microglia activation, and the reduction of pro-inflammatory cytokines and general AD biomarkers [249]. Anticipated results of the phase III trials highlight the slowing or small amelioration of cognitive parameters in **Simufilam**-treated patients with mild-to-moderate AD [250]. Repurposed molecules such as **cromolyn** and **ibuprofen** were proposed in combination and the resulting product (**ALZT-OP1**, phase III) was proven to target microglia by inducing phagocytosis and reducing Aβ levels [251]. Clinical studies were completed (NCT02547818), but no outcome has been published yet. Triggering Receptor Expressed on Myeloid cells 2 (TREM2) is a microglial transmembrane receptor associated with important functions in neuroinflammation, whose disturbances were associated with AD progression [252]. mAbs activating TREM2 were proposed such as **AL002** exhibiting promising results in animal models [253]. Phase II clinical trials are ongoing (NCT05744401). **TAK-920/DNL919** is another antibody targeting TREM2 by modulating its expression via microglia function improvement [254]. The phase I trial in healthy volunteers is ongoing (NCT05450549). Recently, the potential of targeting microglia-induced inflammation via selective CB2 receptor agonists was explored [255,256]. Studies in animal models underline a reduction of inflammation, Aβ clearance, and cognition/memory improvement [255,256]. Phase II clinical studies are ongoing or imminent [257].

### 5.2. Mitochondrial Cascade Hypothesis and Therapeutics

#### 5.2.1. Mitochondrial Cascade Hypothesis

The relation of mitochondrial dysfunction to AD led to the mitochondrial cascade hypothesis [258,259]. Decline in mitochondrial function which induced decreased ATP levels, ROS production increase, and oxidative phosphorylation were highlighted in AD patients [260]. Mitochondrial dynamics, mitophagy, number of mitochondria, and their transport along the axon were also found to be altered [261].

Since the mitochondrial dysfunction appears very early it was hypothesized to be the primary factor inducing Aβ deposition and NFTs formation in late-onset sporadic AD cases [258]. Mitochondrial dysfunctions (e.g., ROS increase) promote Tau hyperphosphorylation and aggregation. Meanwhile, Tau pathology has a negative impact on the mitochondrial axonal transport, mitochondrial dynamics, and function [262]. Moreover, Aβ has toxic effects on mitochondrial basic functions such as respiration and ATP production [263]. Conversely, mitochondrial-derived ROS species promote amyloid formation [264]. Thus, the reversal of mitochondrial function impairment seems to be a viable approach to contrast neurodegeneration [265].

#### 5.2.2. Targeting Oxidative Stress and Mitochondrial Dysfunction

**Figure 9:**
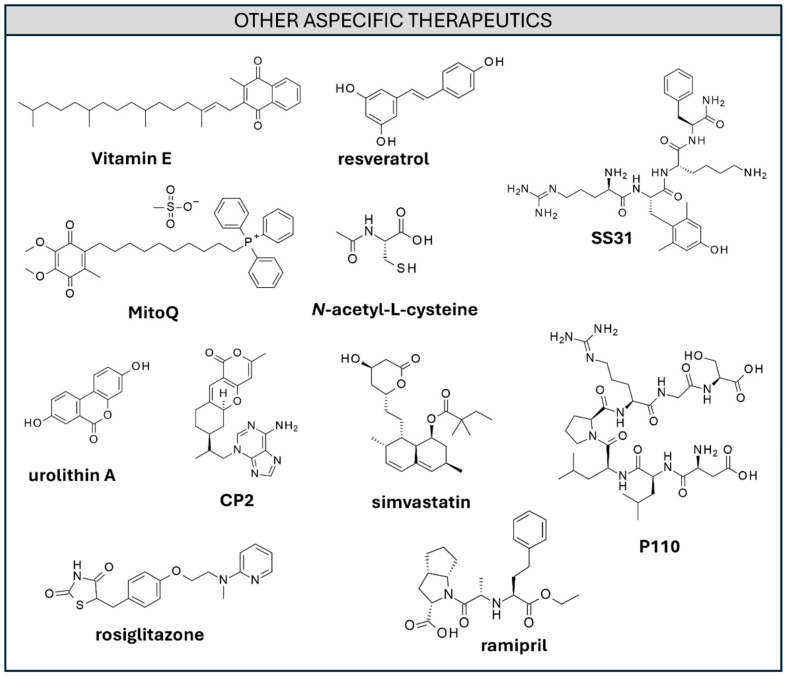
Chemical structure of aspecific therapeutics exploited in AD [1].

The potential of natural antioxidants as AD-preventing agents and in AD treatment has been largely explored (Figure 9), both in the form of dietary supplements and as monotherapies [266,267,268].

Important examples of this class of candidates are represented by **Vitamin E** [269], **Selenium** [270], **Flavonols** [271,272,273,274], and **Resveratrol** (Figure 9) [275,276,277].

**N-acetyl-L-cysteine** (**NAC**) (Figure 9) was shown to enhance GSH levels and is a naturally occurring tripeptide which acts as a ROS scavenger whose levels are lowered in the presence of AD [278]. Moreover, administration of **NAC**-loaded nanoparticles was shown to prevent microglia activation and reduce Aβ levels in animal models [279].

Mitochondrial oxidative stress was targeted by the combination of a mitochondria interacting group (tri-phenyl phosphine) and an antioxidant (hydroquinone), resulting in the drug candidate **MitoQ** (Figure 9) [280]. In mouse models, **MitoQ** was shown to reduce Aβ, oxidative stress, neuroinflammation, and alleviate cognitive impairment [280].

Mitochondrial damage results in an abnormal increase in mitochondrial fragmentation (fission) and biogenesis. **SS31** (Figure 9), a tetrapeptide, was proved to inhibit mitochondrial fission and reduce sAβ, leading to ameliorated synapsis function in animal models [281]. **P110** peptide (Figure 9) was instead designed to disrupt the interaction between dynamin related protein 1 (Drp1) and its mitochondrial adaptor fission 1 (Fis1). **P110** treatment was proven to decrease mitochondrial fission, relieve oxidative stress, and have a positive impact on Aβ deposition in preclinical studies [282,283]. Targeting mitophagy by means of mitophagy inducers such as **urolithin A** (Figure 9) may represent a promising strategy to combat AD. The selective degradation of defective mitochondria, in fact, was shown to diminish Aβ levels, reduce neuroinflammation, and abolish AD-related tau hyperphosphorylation. Prevention of cognitive impairment and even memory impairment reversal were highlighted in animal models [284].

Partial inhibition of mitochondrial complex 1 was attempted by the design of small molecule **CP2** (Figure 9), resulting in a favorable metabolic reprogramming of the cell. Ameliorated cognitive and behavioral profiles were also highlighted [285].

### 5.3. Vascular Hypothesis and Therapeutics

#### 5.3.1. Vascular Hypothesis

The vascular hypothesis [286] arose from the observation of several changes in the cerebral vascular system of AD patients. The revealed modifications include changes in the capillary architecture, BBB damage, reduced cerebral blood flow, glucose metabolism, and oxygen utilization [286,287], which are hypothesized to be involved in AD pathogenesis [286]. Supporting the vascular hypothesis, pre-existing cardiovascular diseases are established risk factors for AD development, [288]. The presence of atrophy of the vascular system in the brain [289] and the tendency of capillaries to exhibit ruptures and bleeding [290] were observed in AD patients. The decreased cerebral blood flow triggers Aβ production via BACE1 upregulation and promotes Tau hyperphosphorylation [291]. Low blood perfusion induces mitochondrial malfunction, leading to ROS generation [292]. However, the role of vascular risk factors in AD development remains unclear; not all patients with such risk factors develop AD. The scarce success of treatments targeting vascular diseases to stop AD progress has led to underlining the necessary presence of other factors to trigger AD development [293].

#### 5.3.2. Therapeutics for Vascular-Based Diseases

According to the vascular hypothesis, several drugs used to treat cardiovascular diseases were evaluated as potential treatments for AD. Statins are a very commonly prescribed medication used to reduce cholesterol levels in the blood. Clinical studies assessing the effect of AD cognitive decline reported mixed results. Recent meta-analysis and studies showed the amelioration of certain cognitive indicators (Mini-Mental State Examination, MMSE) upon statins administration in the short term [294] and after three years of treatment [295]. Several other meta-analyses, however, highlighted the lack of evidence for the pro-cognitive effect of statins [296] in randomized controlled trials. Two clinical studies highlight the detrimental effect of **simvastatin** (Figure 9) on cognitive abilities [297]. Most studies and reviews are cautious in proposing statins as beneficial agents to improve AD [298,299]. To date, no clinical trial involving statins has led to their approval for AD treatment. As an example, **simvastatin** testing in AD clinical trials failed to reduce AD biomarkers in the cerebrospinal fluid [300]. **Antidiabetic** medications such as **rosiglitazone** (Figure 9) were also evaluated towards AD. While beneficial effects of **rosiglitazone** were observed in preclinical studies, the compound posed inactive results in clinical models [93,301,302]. **Antihypertensive** medications such as **ramipril** were evaluated based on the higher burden of AD symptoms in the presence of hypertension [303]. Unfortunately, **ramipril** did not succeed in a pilot clinical trial [304]. The use of angiotensin receptor blockers highlighted a limited pro-cognitive effect in AD patients (9.4%) [305], while angiotensin-converting enzyme inhibitors (ACE-Is) exhibited less efficacy, possibly due to low BBB penetrance. No clear scenario on the use of renin-angiotensin system (RAS)-targeting antihypertensive drugs on cognitive decline in AD has emerged [306].

## 6. FDA-Approved Drugs for Alzheimer’s Disease

**Figure 10:**
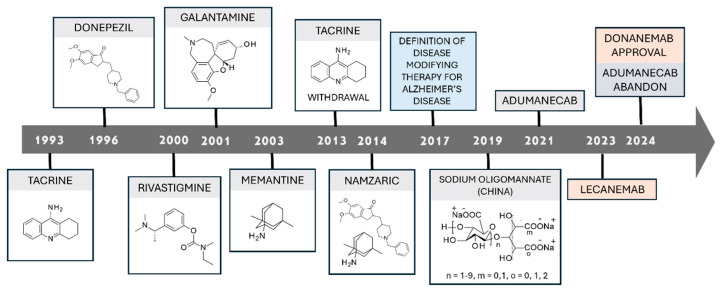
Timeline of the approval and withdrawal of AD treatments [1,307].

Extensive effort was applied to the development of AD treatment, leading to the design and (pre)clinical evaluation of a plethora of candidates. However, only a few compounds reached the market. An outline of the most relevant milestones in AD drug development over the years is given in Figure 10.

Initially, small molecules targeting AChE were proposed [308,309,310,311,312,313], such as **tacrine** in 1993, then **donepezil**, **rivastigmine**, **galantamine**, as well as the NMDAR antagonist **memantine** [314]. In 2014, a dual-acting compound (**namzaric**) was proposed, based on its AChEI and NMDAR antagonist behavior. In 2017 the definition of “disease modifying therapy” arose [315], inspiring, in 2021 and later, the design of anti-Aβ monoclonal antibodies. An overview of the cited medication is given in Table 1.

In addition to FDA-approved drugs, in 2019 an oligosaccharide extracted from marine algae (**sodium oligomannate**, or **GV-971**, Table 1 entry 7) was conditionally approved in China [321]. While the mechanism of action of such therapeutics is still under elucidation, clinical data highlights a good tolerability profile and a certain efficacy [317]. Phase IV clinical trials (NCT05181475 and NCT05058040) are expected to furnish more information by 2025.

Details of the aforementioned most relevant drugs developed to contrast AD are discussed as follows.

### 6.1. AChEIs-Approved Drugs

The AChEI **Tacrine** (Table 1 entry 1) was the first drug approved for AD treatment [308]. A few years ago (2013), it was withdrawn from the market mainly because of its hepatotoxicity [322]. More recently, the **tacrine** scaffold was re-evaluated and modified to avoid liver toxicity issues [323], but no approval has been obtained so far.

**Donepezil** [324,325] (Table 1 entry 2) is a selective and reversible AChE inhibitor. The FDA approved **Donepezil** in 1996 for the symptomatic treatment of mild, moderate, and severe AD [326]. The most common side effects are nausea, vomiting, and diarrhea [327]. Again, **Donepezil** was shown to be inefficacious in altering AD progression [325]. **Rivastigmine** (Table 1 entry 3) was approved in 1997 for mild-to-moderate AD. It acts as a reversible AChE and butyrylcholinesterase [328] inhibitor. However, due to its higher AChE inhibition time with respect to **donepezil**, **rivastigmine** is classified as a pseudo-irreversible AChEI [311]. The most common side effects include gastrointestinal disturbances [329]. **Galantamine** (Table 1 entry 4) is another reversible AChE inhibitor approved by the FDA in 2001 for the treatment of mild and moderate AD stages. In addition to the AChE inhibition, **Galantamine** acts as positive allosteric modulator of the nicotinic receptors [328,330]. **Galantamine** is also a ROS scavenger, acting as a protecting agent from apoptosis [331]. The main side effects are nausea, cramping, salivation, and vomiting [332]. Additionally, musculoskeletal, respiratory, and cardiovascular conditions may arise [333]. It cannot be considered as a disease-modifying drug [334].

A review on the efficacies of AChEIs was carried out in 2021 [335], highlighting the low efficacy of this class of drugs in ameliorating cognitive impairment and behavioral symptoms. The combination of AChEIs and **memantine** was proven to moderately enhance the efficacy with respect to monotherapy [336]. A combination of **donepezil** and **memantine** was approved for moderate and severe AD (**Namzaric**, Table 1 entry 6) [337].

### 6.2. NMDAR Antagonists as Approved Drugs

As an NMDAR antagonist, **memantine** (Table 1 entry 5) regulates the glutamatergic system, thus influencing the correlated calcium signaling. More precisely, **memantine** acts as a selective, moderate-affinity and uncompetitive E-*N*-methyl-d-aspartate receptor blocker [338], inhibiting the excess of calcium from entering neuronal cells while allowing physiological glutamate activity [339,340].

The selectivity of **memantine** towards the E-NMDAR (extra synaptic) with respect to the S-NMDAR (synaptic) represents a favorable factor to recover physiological glutamate balance. While E-NMDAR overstimulation induces the activation of pro-death pathways, prolonged S-NMDAR and AMPAR activation causes their desensitization and internalization, leading to the loss of glutamatergic signaling [341].

**Memantine** was shown to be safe and tolerable, with an adverse effect frequency comparable to placebo [342]. **Memantine** was shown to slow down cognitive decline in various clinical studies when considering moderate-to-severe AD [343]. On the other hand, no evidence of efficacy was highlighted in earlier stages of the disease [343,344]. **Memantine** was also shown to antagonize 5-HT_3_ receptors at therapeutic NMDAR blocking concentration [345]. Despite the presence of several studies supporting the potential of **Memantine** (especially in preclinical models), Kuns et al. report that in clinical practice the response to **Memantine** was mild, leading to limited benefits [346].

### 6.3. Monoclonal Antibodies

To date, three monoclonal antibodies directed towards Aβ clearance have been recently approved by the FDA: **Aducanumab**, **Lecanemab**, and **Donanemab** (Table 1 entries 8, 9, 10, respectively) [347].

In detail, **Aducanumab** was approved in 2021, being selective towards protein aggregates with respect to Aβ monomers and binding both soluble and insoluble Aβ [348]. The corresponding complexes are recognized and phagocyted by microglia [349]. Additionally, **Aducanumab** was shown to limit Aβ toxicity by preventing Aβ release from plaques [349]. Pro-cognitive effects of **Aducanumab** were established via cognitive tests, giving encouraging results [349,350]. However, some trials failed to reproduce these positive outcomes [351]. Very recently, Biogen, the company that developed **Aducanumab**, decided to discontinue the development and production of this monoclonal antibody [352] to prioritize the development of the novel mAb **Lecanemab**, approved in 2023 for the treatment of MCI or mild AD dementia [347,353].

**Lecanemab** was shown to bind soluble Aβ aggregates, comprehending oligomers and protofibrils [353]. **Lecanemab** decreases brain Aβ and reduces the presence of several biomarkers [354]. This monoclonal antibody exhibited moderate slowing of cognitive decline in a large-scale phase III clinical trial, involving participants with mild AD [355,356]. No data are available on more advanced stages of the disease [357].

**Donanemab** is a recently approved (2024) monoclonal antibody specifically designed to bind the pyroglutamate Aβ p3-7 epitope, a type of truncated Aβ exclusively found in deposited Aβ [358,359]. Reduction in the levels of AD biomarkers (such as Aβ plaques and plasma p-Tau217) was observed during clinical trials [360]. A certain slowing of cognitive decline was observed in clinical trials concerning the early AD phase [361].

Anti-Aβ monoclonal antibodies were proven to efficiently exert an Aβ clearance function, being designed as a disease-modifying therapy for AD at a conceptual level [362,363]. Nevertheless, the clinical outcome was less convincing, resulting in limited efficacy in several clinical trials and real-life situations [363].

## 7. Therapeutic Approaches: Lights and Shadows

In this study, we reported neurotransmitter- or ion-based AD pathogenesis hypotheses as well as peptide-based ones and aspecific pathways. None of them can be considered an exhaustive and complete explanation of the AD etiology and the related biological processes. Targeting receptor-based systems such as the cholinergic pathways and NMDARs is controversial, as it has been debatably proved to adopt the role played by peptide-based deposits (Aβ and tau plaques).

Cognitive impairment due to the manipulation of the cholinergic system was observed in animal models of AD [364,365]. However, despite the large amount of data supporting the cholinergic hypothesis, AChEI proved poor efficacy in AD patients [366]. Thus, it was attempted to combine the pro-cholinergic dual inhibition action of the AChE and other enzymatic targets with other favorable pharmacological effects. An example previously mentioned is the hybrid AChE/MAO-B inhibitor **Ladostigil** showing its good tolerability but lack of efficacy in slowing down AD progression [59] (NCT01429623). The same strategy was applied to guide the design of dual AChE/GSK-3β inhibitors and dual AChE/PDE4D [62]. On the other hand, the design of allosteric modulators on AChR subtypes [63,64,65,66] was explored to confer higher selectivity to the compound.

Targeting allosteric sites rather than orthosteric ones is expected to confer higher selectivity to the compound, given the lowest degree of residue conservation observed for allosteric sites. In addition, receptor desensitization upon repeated doses associated with ligands binding the orthosteric site can be reduced. α7 nAChR positive allosteric modulators exhibited promising pro-cognitive effect in animal models [67,68,69,70]. Conversely, other clinical studies suggested unclear efficacy of this class of compounds [71].

**Memantine** is also an antagonist of nACh and 5-HT_3_ receptors, suggesting once again the design of multi-target molecules. Indeed, memantine hybrids were recently proposed as potential disease-modifying agents for AD, as they are expected to target multiple AD typical processes [367]. **Memantine** is also included in the therapeutic strategies targeting the Ca^2+^ signaling disruption hypothesis. The compound exhibited promising results in clinical studies justifying its approval for AD [368]. On the other hand, limited efficacy was highlighted over time [80]. Clinical studies concerning Ca^2+^ channels gave mixed results [369]. The therapeutic potential of calcium modulators is moderated by the ubiquitous presence of Ca^2+^ in cellular processes, which may be the cause of side effects arising.

Examples of Aβ targeting strategies involve monoclonal antibodies, with Aβ clearing function [370] and inhibitors of β-site amyloid precursor protein cleaving enzyme 1 (BACE1) being thie enzyme involved in Aβ production [371]. The design of BACE inhibitors led to compounds exhibiting promising effects on preclinical and phase I–II clinical trials. On the other hand, significant side effects were reported [217]. In addition, clinical trials involving compounds targeting Aβ failed in ameliorating or stopping cognitive impairment associated with AD, possibly underlying that Aβ accumulation is not sufficient to cause AD [49].

Recently, the amyloid hypothesis was further revised, decreasing the weight of Aβ deposition and increasing the weight of stochastic factors [372], switching the cause of AD to soluble Aβ oligomers in place of plaques [373] and introducing higher attention to interactions with other pathology-associated mechanisms [374]. Three anti-Aβ mAbs were recently approved by the FDA for clinical use. Early data concerning mAbs as potential AD-modifying therapeutics exhibited only moderate efficacy in slowing the disease, but were not shown to reverse AD pathology, possibly questioning the amyloid hypothesis itself [375]. Thus, the clinical efficacy of Aβ directed monoclonal antibodies is controversial as limited improvement of cognition is observed while adverse effects are relatively frequent [376,377].

Furthermore, the development of several kinase inhibitors was evaluated as a therapeutic approach. In particular, GSK-3β inhibitors such as **Tideglusib** and Fyn kinase inhibitors such as **AZD0530** were studied, unfortunately with poor therapeutic efficacy [213] or no effects on cognition decline [214].

Isoform selective JNK3 kinases inhibitors and hybrids between an AChE inhibitor and a Calcium channel blocker (**SCR1693**) led to more promising results in terms of ameliorated cognitive performances [215] and Aβ reduction in vitro [216], respectively. Additionally, multi-kinase inhibitors such as **LDN-193594** showed efficacy in the treatment of cognitive impairment and reduction in hyperphosphorylated tau levels [217].

In addition, monoclonal antibodies such as **ABBV-8E12** targeting tau proteins were proved to reduce Tau protein in Cerebrospinal fluid but did not lead to a reverse of the cognitive impairment [378] (NCT03712787).

A tau-related strategy led to the evaluation of **Epothilone D** in mouse models, resulting in improved cognitive outcomes [221,222,223].

Despite the encouraging results in reducing Tau and Aβ burden, the overall clinical experimentation of Tau-directed monoclonal antibodies was largely unsuccessful [379] and lacking efficacy. Conversely, the PROTAC technology proved to be advantageous; chimeric peptides led to candidates endowed with efficacy in lowering Tau levels [236] and significant pro-cognitive effects [237].

Beyond the cited therapeutic approaches, aspecific treatments have been considered, such as agents to contrast neuroinflammation or mitochondria dysfunction.

Major limitations of the inflammation hypothesis lay in the aspecificity of the inflammation response, which is common to many other pathological conditions. It is also not clear whether inflammation is effectively a cause which contributes to AD development or is instead the consequence of neuronal damage. Nevertheless, a few pharmacological strategies targeting neuroinflammation were proposed [257]. In particular, targeting microglia-induced inflammation via selective CB2 receptor agonists was reported as advantageous [255,256].

The decline in mitochondrial function, causing diminished ATP levels and an increased ROS production, was highlighted in AD patients [258,259]. Thus, products with antioxidant action, as well as anti-inflammatory agents and habits reducing ROS levels (exercise, diet, etc.) were proposed as therapeutic interventions for AD [380,381]. However, further studies are required to better clarify the mitochondrial alterations in relation to AD onset and progression, and to better validate the potential of antioxidants for AD treatment.

In addition to the aforementioned drugs, additional treatments are suggested to treat behavioral and psychological symptoms associated with AD, including orexin receptor antagonists, antipsychotics, antidepressants, and anticonvulsants.

**Suvorexant**, an orexin receptor antagonist, is utilized to treat insomnia in mild-to-moderate AD patients [382]. Such use is associated with multiple side effects such as impaired alertness and motor coordination, worsening depression or suicidal thinking, sleep paralysis, and respiratory function problems [383].

The use of atypical antipsychotics (**Risperidone**, **Olanzapine**, **Quietiapine**, and **Apiprazole**) can be considered to treat agitation, psychosis, and aggression when the symptoms represent a danger for the patient or caregiver. The treatment with antipsychotics should be limited to a few months, as longer therapy was shown to be ineffective [384] and could increase the risk of death in the long term [385].

**Brexpiprazole** is a partial agonist of dopamine D_2_ and serotonin receptor 5HT_1a_, and an antagonist of the serotonin receptor 5HT_2a_ [386]. As an atypical antipsychotic, **Brexpiprazole** has been approved to treat AD-associated agitation [387,388,389]. The use of **Brexpiprazole** is associated with several side effects (weight gain, sleepiness, dizziness, cold symptoms, and restlessness) as well as an increased risk of death when administered to patients with dementia-related psychosis [383].

The use of selective serotonin reuptake inhibitors (SSRIs) was proved to induce a moderate pro-cognitive effect in AD patients [390]. However, results on their efficacy and safety profile depict a contradictory scenario [390,391,392,393,394]. A few studies proposed the possibility that AD-associated depression may exhibit different features with respect to non-AD-associated depression, thus making classical antidepressant treatment inefficacious [392].

Anticonvulsants such as **Carbamazepine** can also be considered for the treatment of AD-associated agitation, aggression, and hostility. However, its lower tolerability as well as interactions with other drugs makes it a less attractive option with respect to SSRIs [395,396]. Psychostimulants such as **methylphenidate** was proven to decrease the severity of apathy in AD patients, exhibiting good tolerability profile [397].

## 8. Medicinal Chemist Opportunities and Challenges

The multi-faceted character of AD often requires a combined approach to develop effective pharmacological strategies targeting AD, going beyond the targeting of amyloid beta or of a single hypothesis but integrating multiple pathways of interventions. Indeed, the individuation and pharmacological modulation of new targets involved in the multiple biochemical pathways altered in AD may help in the optimization of an explorative therapeutic strategy. A few hints for rational approaches by medicinal chemists in the search for putative effective ligands in AD are described as follows.

### 8.1. Mutated Proteins Interventions

As previously described, genetic factors are thought to be in AD pathogenesis. Among them, APP gene mutations, as well as PSEN-1 gene mutations are reported as involved in AD forms. In the first case, mutated APP enzymes lead to an improved Tau quantity, while mutated PS-1 proteins can enhance the production of toxic amyloid peptide. Furthermore, polymorphisms of ApoE-ε4 result in less efficient proteins in the degradation of soluble Aβ. Conversely, other ApoE isoforms work to remove Ab plaques.

Since APP interaction with secretase proteins is required to derive amyloid peptides, the design and evaluation of APP-interfering modulators could be hypothesized, with the aim of developing small molecules that are able to prevent the proteolytic cleavage turning in the Ab amyloid peptides and fibrils. Approximately 25 mutations in APP are pathogenic and cause AD, many of which located in the transmembrane helix domains (TMDs). The APP structure has been investigated by several research groups [398], and biophysical studies were conducted to explore any structural or folding variations due to specific APP mutations affecting recognition by secretases [399,400]. As a consequence, there are data available at the protein data bank, enabling computational studies to be pursued to probe protein pockets for the design of modulators. A deep analysis and comparison of the available APP mutants could allow us to identify the most variable protein regions, in terms of ability to be exposed to the putative secretase recognition in comparison to the WT APP. The results should be compared with the known severe AD forms in order to map druggable protein regions for the design of modulators.

The choice of PS1 modulators can also be useful to avoid the PSNE-1 mutation consequences in AD. Research studies reported mesenchymal stromal (stem) cells (MSCs) as models of early-onset familial AD (FAD) [401]. MSCs with the FAD mutation PSEN1 E280A and wild-type (WT) PSEN1 from umbilical cords were cultured and characterized for their transdifferentiation into cholinergic-like neurons (ChLNs). PSEN1 E280A ChLNs, but not WT PSEN1 ChLNs, exhibited increased intracellular soluble amyloid precursor protein (sAPPf) fragments and extracellular Aβ42 peptide, as described for FAD caused by mutant PSEN1. In addition, PSEN1 E280A ChLNs presented oxidative stress. Very recently, authors investigated whether the phosphodiesterase type 5 inhibitor **Sildenafil** modifies the phenotype of ChLNs, bearing the PSEN-1 E280A mutation [402]. To this purpose, WT ChLNs and ChLNs expressing PSEN 1 E280A were left untreated or treated with the PDE5 inhibitor. While sildenafil treatment caused no alterations in APP metabolism in WT neurons, A**β** accumulation decreased in mutant ChLNs, if compared to the untreated mutant cells. In addition, **Sildenafil** did not affect Tau levels in WT ChLNs but reduced them in ChLNs harboring the PSEN 1 E280A mutation with respect to the untreated mutant cells. As a result, the compound was proposed as a functional neuronal enhancer. To further investigate the potential therapeutic role of this compound as a PS-1 modulator, the PS-1 E280A mutant should be modeled in silico and compared to the experimental data of the WT PS-1 in order to enlighten the corresponding putative druggable pockets. PS-1 E208A correctors could be screened via computational methods and molecular dynamics simulations, prior to chemical synthesis and biological evaluation. In addition, since PDE5 is highly upregulated in AD patients [403], the efficacy of further PDE5 inhibitors series could be evaluated also in other AD forms. Interestingly, cAMP-selective PDE inhibitors, such **cilostazol**, have been reported as neuroprotective agents based on their anti-inflammatory properties [404]. In particular, **cilostazol** caused an elevation of cAMP, which may be responsible for the drug-induced upregulation of SIRT1. This effect is suggested to contribute to the observed anti-inflammatory, antioxidant, and antiapoptotic activities of **cilostazol** also at the central nervous system [404]. More recently, **cilostazol** proved to mitigate neuroinflammation, also counteracting mitochondrial dysfunction in encephalopathy models [405]. Based on the above, the design of dual-acting PDE3/5 inhibitors could be attempted.

With regard to ApoE-ε4 polymorphisms, among subjects without A**β** deposition, APOE-**ε**2 carriers have better-maintained cognitive functions if compared to APOE-**ε**4 carriers [406]. Moreover, in the presence of AD pathology, APOE-**ε**2 carriers present slower cognitive decline, while APOE-**ε**4 carriers experience faster decline compared with APOE-**ε**3 homozygotes. In a medicinal chemistry point of view, designing selective APOE-**ε**4 modulators to prevent AD and/or diminish symptoms could be attempted, relying on structural information of the biological target.

### 8.2. Neurotrasmitter- and Ion-Based Interventions

Since AChE impairs the Ach levels and enhances PS-1 expression, the choice of dual-acting compounds exhibiting inhibitory ability towards the two proteins could represent a feasible strategy. Indeed, experimental data of both the two biological targets are available, allowing structure-based studies as well as numerous series on related inhibitors. This piece of information could be exploited to develop the main pharmacophore features to be merged in putative dual-acting derivatives. In the scenario of hybrid compounds, we previously briefly reviewed a number of studies suggesting AChE/GSK-3β inhibitors or dual AChE/PDE4D. This kind of approach could be enhanced by combined structure- and ligand-based studies, as described for dual-acting AChE/PS-1 modulators. Notably, while dual AChE/PDE4D could result in improved ACh levels and anti-inflammatory responses, AChE/GSK-3β or AChE/PS-1 could be efficient in reducing A**β** levels.

Targeting GLT-1 and NMDARs has been attempted, with the final aim of reducing glutamate excitotoxicity. This event results in the major entrance of calcium into neurons and ROS production increase, and also in tau hyperphosphorylation induction via kinase stimulation. While GLT-1 modulators gained modest results in clinical trials, recently NMDARs inhibitors gained attention and guided the design of several series of memantine analogs. This approach sounds worthy of further efforts towards novel derivatives to mitigate neuroinflammation, and conceivably to design multi-target compounds also exhibiting the AChE inhibitory ability.

With regard to the serotoninergic hypothesis, the role of 5-HTRs in cognition needs to be better clarified. Based on the previously reported information, 5-HT_4_ and/or 5-HT_7_ agonism gave promising results in clinical data, supporting new studies for the development of selective ligands. Conversely, the effectiveness of 5-HT_6_ antagonists in clinical trials proved to be modest. In 2022, the electron microscopy data of 5-HT_4_ and of 5-HT_7_ became available (PDB code = 7XT9; PDB code = 7XTC) [407], paving the way for the rational design of new ligands. In 2023, the structural information of 5-HT6 (PDB code = 7YS6) [408] also offered the opportunity to better investigate putative binding pockets to design novel antagonists.

### 8.3. Calcium Signaling Modulator Interventions

Among plausible interventions to modulate calcium levels, targeting NMDARs or RyRs could be attempted. Furthermore, S1Rs are widely expressed throughout the CNS and modulate neuron intracellular calcium levels, leading to changes in neurotransmitter release and neuronal activity [409]. Thus, as previously shown for **Anavex 2-73**, ligands interacting with S1Rs represent a promising approach in drug design for the treatment of AD. Notably, the FDA drug donepezil also features the S1R binding ability [410]. In this perspective, dual AChEIs and S1R agonists should be designed and scouted for models of cognitive decline.

### 8.4. Peptide-Based Level Interventions

We reported amyloid-targeted treatments including APP cleavage and Aβ aggregation inhibition. During the last few years, different series of **γ**-secretases inhibitors have been proposed, even if with safety limitations. Accordingly, allosteric inhibitors have been suggested. With regard to Aβ aggregation inhibition, peptidomimetics targeting A**β** were proposed, also relying on immunotherapy approaches.

The design of optimized compounds modifying the APP proteolytic event can be managed by the secretase inhibitors design or by the PS-1 unit one. Recently, the electron microscopy analysis of human g-secretase in complex with the inhibitor **Avagacestat** (PDB code = 6LQG) [411] has been reported, giving pivotal information for the design of further new molecules. Indeed, structural analyses revealed a set of shared interactions and contact regions for inhibitor and modulator recognition to be exploited for the future development of substrate-selective inhibitors. More recently, authors reported cryo-electron microscopy structures of human **γ**-secretase bound to five clinically tested inhibitors (**RO4929097**, **crenigacestat**, **BMS906024**, **nirogacestat**, and **MK-0752**) [412]. All of them occupied the substrate-binding site of PS-1 even if exhibiting different protein-contact interactions based on their chemical structure. A comparison of the main chemical features of the compounds should be attempted to derive the main pharmacophore features for the optimization of a new series; while **RO4929097** and **crenigacestat** displayed a tricyclic main core, bearing a flexible chain, the other ones were bicyclic or monocyclic derivatives tethered to electron-rich aliphatic substituents. Based on the overall flexibility of the aforementioned inhibitors, molecular dynamics simulations could be beneficial in interpreting the enzyme-ligand binding event.

In the search for Tau phosphorylation modulators, kinase inhibition can be managed via computational methods. A few X-Rays of the crystallographic data of GSK-3**β**, in complex with inhibitors, are available (PDB code = 3SAY) [413], supporting structure-based design strategies of new compounds.

## 9. Innovative Strategies and Future Perspectives

Extensive research effort in recent decades was applied to the design of therapeutic agents for Alzheimer’s disease, as previously reviewed. The scarce success of such candidates is inserted into a larger frame of uncertainty regarding the pathogenic mechanism(s) determining the disease. The elucidation of the biochemical pathways to be targeted for intervention is strongly required to allow a rational approach of drug design and development.

However, several obstacles are present to achieve such results, such as the extreme complexity and interconnection observed among different pathogenic pathways. Then, preclinical translation to clinical tests is also a limiting factor in AD therapeutic attempts, mainly due to current AD animal model limitations [414]. Nevertheless, continuous effort in optimizing existing experimental models [415] or introducing new ones has been applied, leading to novel tools such as organoids and patient-derived AD cells for screening, in place of mouse models for AD [416,417].

In addition to the most studied mechanisms herein reviewed, several emerging hypotheses are likely to furnish more insights on AD pathogenesis. A few examples include the infectious hypothesis [418,419], the iron dyshomeostasis hypothesis [420], lipid invasion [421], the Carnitine Palmitoyl-Transferase 2 Cascade Hypothesis [422], studies on the role of neurotropins [423], and gut microbiota [424], among others. Such theories are often neglected or underconsidered in general AD studies but may be included in a holistic vision of the disease to highlight previously uninvestigated links and concepts. Thus, future intervention on newly discovered pathogenic mechanisms may lead to improved therapeutic approaches.

In particular, the multi-target approach [425] is regarded as an attractive avenue for the future development of AD treatment(s), combining multiple pharmacological actions within a single molecule and influencing multiple pathways at a single time. Such an approach possesses great potential in the case of multifactorial pathologies, where it can be argued that various processes are involved in the genesis of the disease. Multitarget therapy exhibits several advantages with respect to combination therapy, as the risk of adverse effects and drug resistance development is significantly lowered [426]. Moreover, drug–drug interactions influencing the drug bioavailability are avoided [427], and often better compliance is achieved due to a simpler therapy regimen [428].

Gene-therapy development also represents a promising strategy for AD treatment, despite the presence of several challenges such as the regulatory and commercial aspects, as well as the need for efficient and secure gene delivery systems [429]. Gene therapy consists of gene manipulation including the deletion, silencing, or editing of faulty genes and insertion of healthy genes [430]. Carriers (mainly viral carriers) are used as a delivery strategy for the gene material. A few promising results were highlighted for complex neurological diseases such as AD [431,432], as low bioavailability at the CNS still represents a critical aspect in anti-AD drug development [433].

The growth of the nanobiotechnology field represents an interesting opportunity to enhance brain penetrance of AD drug candidates. Nanoparticles (NPs) facilitate the passage of therapeutics through the BBB, with relevant potential both in the diagnostic and therapeutic areas [434,435]. PROTACs [436] and protein–protein interaction modulators [437] are also developing strategies in AD research. More recently, photobiomodulation (PBM) based on specific light wavelengths to regulate cellular metabolism, signal transduction, and gene expression has been reported as a promising opportunity to enhance AD cognitive function [438]. This study underlined the PBM ability to improve BBB integrity impairing Aβ pathology in AD mice models.

Improvements in AD diagnosis, whose technical limitations have been extensively reviewed [417], should be achieved. In particular, a subjective use of neurophysiological scales, the expensiveness or low compliance of diagnostic procedures, and once again questionable adequateness of currently used diagnostic biomarkers are regarded as improvable factors in the AD diagnostic process. It has been shown that such biomarkers [439] do not always correlate with disease severity, do not cover the full spectrum of AD pathology [440], and are highlighted in other brain diseases [441]. Recently, the search for periphery biomarkers for the preclinical diagnosis of AD has been investigated [417] and screenings were suggested for high-risk populations.

Additional measures aiding research proficiency in addressing such a complex matter have been proposed. A few examples [442] include a global effort in the sharing of data, developing accessible diagnostic tools and affordable therapeutics, correcting the management of risk factors and developing AD-focused educational programs, as well as new clinical trial practices (e.g., patient stratification and precision medicine model use [443]). Finally, technologies such as computer-aided drug design (CADD) and artificial intelligence (AI) are also thought to produce relevant outcomes at the drug design level [444,445].

## 10. Conclusions

To date, the FDA approved four small-molecule drugs for the treatment of Alzheimer’s disease. Three of them (**Donepezil**, **Galantamine**, and **Rivastigmine**) can be classified as acetylcholinesterase inhibitors (AChEIs), while the fourth (**Memantine**) is an *N*-methyl-d-aspartate receptor (NMDAR) antagonist [328]. In addition, a few monoclonal antibodies (mAbs) were recently approved [446]. Non-pharmacological interventions such as care and quality of life are commonly prescribed as first-line intervention to prevent cognitive decline; exercise, diet attention, and vascular- or metabolism-related disease prevention should be beneficial to limiting AD risk factors.

The current AD scenario highlights a complex intricacy of processes in which it is not easy to distinguish the key factor(s) from ancillary factors, or to elucidate the synergistic effect that may arise from concomitant pathogenic signs. From a therapeutic perspective, this is reflected in difficulties in finding disease-modifying strategies, which results in the lack of adequate/resolving treatment for AD patients. Further efforts to clarify druggable biochemical pathways are still needed to guide the drug design process.

## Figures and Tables

**Table 1:** List of the approved (and withdrawn) anti-AD drugs. Approval year (appr. year), references (ref.), and side effects are reported.

Entry n.	Drug Name	Class	AD Stage	Appr. Year	Withdrawn	Side Effects	Administration	Ref.
1	Tacrine	AchEI	Mild-to-moderate	1993	Yes (2013)	Hepatotoxicity	oral	[308,309]
2	Donepezil	AchEI	Mild, moderate, severe AD	1996	No	Nausea, Vomiting, Diarrhea, Fatigue	Oral transdermal	[310]
3	Rivastigmine	AchEI	Mild-to-moderate	2000	No	Dizziness, Vertigo, Upper respiratory tract infection	Oral transdermal	[311,312]
4	Galantamine	AchEI	Mild-to-moderate	2001	No	Salivation, Bradycardia, Dizziness, Abdominal pain	Oral	[313]
5	Memantine	NMDAR antagonist	Moderate-to-severe	2003	No	Headache, Confusion, Dizziness, Urinary infections, Constipation, Somnolence, Agitation	Oral	[314]
6	Namzaric	AChEI NMDAR antagonist	Moderate-to-severe	2014	No	Dizziness, Agitation, Confusion, Diarrhea, Nasopharyngitis, Falls	Oral	[316]
7	Sodium oligomannate	Not known	Mild-to-moderate	2019	No	Infections, Gastrointestinal tract disorders, Nervous system problems	Oral	[317]
8	Aducanumab	Anti-Aβ mAbs	MCI, mild AD	2021	No (discontinued after approval)	ARIA ^a^ side effects	Intravenous	[318]
9	Lecanemab	Anti-Aβ mAbs	MCI, mild AD	2023	No	ARIA side effects	Intravenous	[319]
10	Donanemab	Anti-Aβ mAbs	MCI, mild AD	2024	No	ARIA side effects	Intravenous	[320]

^a^ ARIA: Amyloid-Related Imaging Abnormalities.

## Data Availability

Data sharing is not applicable.
